# Is Acute Lower Back Pain Associated with Heart Rate Variability Changes? A Protocol for Systematic Reviews

**DOI:** 10.3390/healthcare12030397

**Published:** 2024-02-03

**Authors:** Gema Sanchis-Soler, Juan Tortosa-Martinez, Sergio Sebastia-Amat, Ivan Chulvi-Medrano, Juan Manuel Cortell-Tormo

**Affiliations:** 1 Department of General and Specific Didactics, University of Alicante, 03690 San Vicente del Raspeig, Spain; gema.sanchis@ua.es (G.S.-S.); sergio.sebastia@ua.es (S.S.-A.); jm.cortell@ua.es (J.M.C.-T.); 2 Health, Physical Activity and Sports Technology (HEALTH-TECH), University of Alicante, 03690 San Vicente del Raspeig, Spain; 3 Department of Physical and Sports Education, University of Valencia, 46010 Valencia, Spain; ivan.chulvi@uv.es

**Keywords:** sciatica, autonomic nervous system, sympathetic nervous system, parasympathetic nervous system, vagus nerve, heart rate variability, back pain

## Abstract

Acute lower back pain (ALBP) is an extremely common musculoskeletal problem. ALBP consists of a sudden onset of short-duration pain in the lower back. However, repeated attacks can make the pain chronic. It can be measured through a self-report scale as well as through physical and physiological evaluations. Heart Rate Variability (HRV) has been used to evaluate the body’s response to pain. However, to the best of our knowledge, no clear consensus has been reached regarding the relationship between both variables and on an optimal protocol for ALBP evaluation based on HRV. The objective of this review is to analyze the relationship and effectiveness of HRV as an instrument for measuring ALBP. Furthermore, we consider the influence of different types of interventions in this relationship. The protocol of this review was previously recorded in the International Prospective Register of Systematic Reviews (number CRD42023437160). The PRISMA guidelines for systematic reviews and PubMed, WOS and Scopus databases are employed. Studies with samples of adults with ALBP are included. This study sets out a systematic review protocol to help identify the relationship between HRV and ALBP. Understanding this relationship could help in designing early detection or action protocols that alleviate ALBP.

## 1. Introduction

Pain can be defined as a sensory perception and alarm mechanism that varies from one person to another and is mediated by sensory neurons (nociceptors), by different nervous system mechanisms, as well as by psychological and social factors [1,2,3]. Pain can be classified based on the pathophysiological mechanisms that produce it, its duration, its etiology or anatomical location [4]. It is, however, sometimes necessary to adopt a broad approach in order to precisely define the origin of the pain and thus implement appropriate therapeutics [5]. Furthermore, since pain is a subjective and intrinsic sensation of the individual, making an accurate measurement is sometimes complex [6].

Such cases include lower back pain. A large share of the population suffers from chronic lower back pain. According to the World Health Organization, lower back pain affected over 600 million people in 2020 and this figure is estimated to exceed 800 million by 2050 [7]. Based on these numbers, chronic lower back pain is the most prevalent musculoskeletal problem across all population ages [8,9]. Not surprisingly, therefore, lower back pain has a very high economic impact. A recent systematic review and meta-analysis analyzing data from high income countries estimated that the average annual direct costs per population for lower back pain ranged from EUR 2.3 billion to EUR 2.6 billion, and the indirect costs ranged from EUR 0.24 billion to USD 8.15 billion. The estimated pooled direct cost per patients were USD 9231, and total costs USD 10,143.1 [10].

Chronic lower back pain is characterized by lower back pain that lasts for more than 12 weeks [11,12]. Despite a variety of risk factors, chronic lower back pain is often caused by repeated episodes of acute lower back pain (ALBP) or lower back pain lasting less than 6 weeks [5]. In their study, after measuring and controlling 5233 subjects, Stevans et al. [13] observed that 32% of patients with ALBP transitioned to chronic lower back pain. This transition appears to be modulated by both complex inflammatory processes and central nervous system alterations [14]. In this way, certain movements or postural changes can provoke the inflammation of musculoskeletal structural elements involved in lumbar spine stability (joints, ligaments, muscles), thus contributing to central and peripheral sensitization [5].

Moreover, both ALBP and chronic lower back pain often cause incapacity for certain everyday activities, including those related to the workplace (absenteeism) [15]. The latter represents a significant economic burden for the health system and for companies whose employees resort to sick leave due to the disability caused by pain [16].

Given the risk of pain chronicity and the high economic costs deriving from it, it is important to diagnose and treat the pain early. To this end, a range of techniques allow the direct or indirect assessment of acute pain. Examples include face-to-face interviews, nerve conduction velocity tests or techniques for detecting variations at the physiological level [6,11,12,13,17]. In fact, in recent years, the use of instruments or technologies that allow for the assessment of pain from a physiological perspective has been proposed. In a recent review, Fernandez Rojas et al. [6] studied the different techniques used to detect pain according to changes in the autonomic nervous system, central nervous system and brain activity. Among the most used techniques we may find heart rate, heart rate variability (HRV), photoplethysmography, electrodermal activity, respiration, electromyography, electroencephalography and magnetic resonance imaging.

Each of these techniques has both advantages and disadvantages [6]. Thus, using protocols that integrate different optimal variables within the same pain measurement procedure may allow for obtaining a more complete and reliable evaluation. To achieve this, it is important to study the efficacy of each of the available variables.

HRV is a physiological parameter that has been proposed as an index of the flexible and adaptive regulation of the nervous system to organize a homeostatic response to diverse types of stressors and environmental contexts [1]. More specifically, HRV represents the change in the time interval between successive heartbeats, and shows the heart’s ability to adapt to changing situations [18], including when a person experiences pain.

Thus, the activation of HRV allows the body to provide an adaptive response to stressors such as pain. In a recent review, HRV was found to be an optimal variable for measuring (sympathetic and parasympathetic) autonomic nervous system sensitivity to nociceptive stimulation [1]. In fact, there seems to be a strong interaction between the nociceptive nervous system and the autonomic nervous system, in both the sympathetic and parasympathetic branches [19,20].

For this reason, some studies have proposed including it as a parameter to consider when evaluating pain and the benefits of certain treatments, taking into account sympathetic balance and the stress levels associated with these types of ailments [17,21]. In this way, Bandeira et al. [22] conducted a review to determine whether patients with chronic low back pain presented HRV alterations. However, only two studies were included in their analysis. Thus, the evidence they obtained was limited. Despite these results, HRV may be an optimal variable to detect acute pain. Considering the tendency of HRV to undergo variations, measurement protocols must be concrete and precise. To this end, conducting the evaluation while the movement that produces the most disabling pain is taking place or during the most acute phases of pain may provide the required information. In parallel, in relation to the treatment of pain, different studies expose the benefits or effects of therapies such as chiropractic or relaxing massages on the functionality of the autonomic nervous system [23,24].

Given that ALBP is regarded as an antecedent risk factor of chronic low back pain, investigating the possible relationship between HRV and ALBP could help to prevent and/or treat this pathology. Therefore, the objectives of this review will be (1) to analyze the possible relationship between HRV and ALBP, (2) to study the feasibility and effectiveness of HRV as an ALBP measurement instrument and (3) to assess the influence of different types of interventions on the relationship between HRV and ALBP.

## 2. Materials and Methods

To ensure maximum review transparency and to reduce possible biases [25], the protocol of this review was recorded in the International Prospective Register of Systematic Reviews (number CRD42023437160). To prepare the protocol, we followed the PRISMA guidelines (Preferred Reporting Items for Systematic Reviews and Meta-Analyses) [26,27].

This methodology establishes the steps and content that a quality systematic review must present. The PRISMA methodology establishes that a systematic review or meta-analysis article must contain the following parts: a title, abstract, introduction, methodology, results, discussion and other information. It should be noted that the methodology must include a flow chart that specifies the process of searching and selecting studies, indicating the reasons for exclusion in each case. In relation to the results, they must contain a summary table of the selected articles as well as an analysis of the risk of bias and quality of the studies.

On the other hand, in the recent update of the PRISMA regulations [27] it is established that articles or reports included in previous reviews, as well as studies identified through other means such as websites, organizations and citation searching, can be considered for the final selection of the studies to be analyzed.

**Figure 1:**
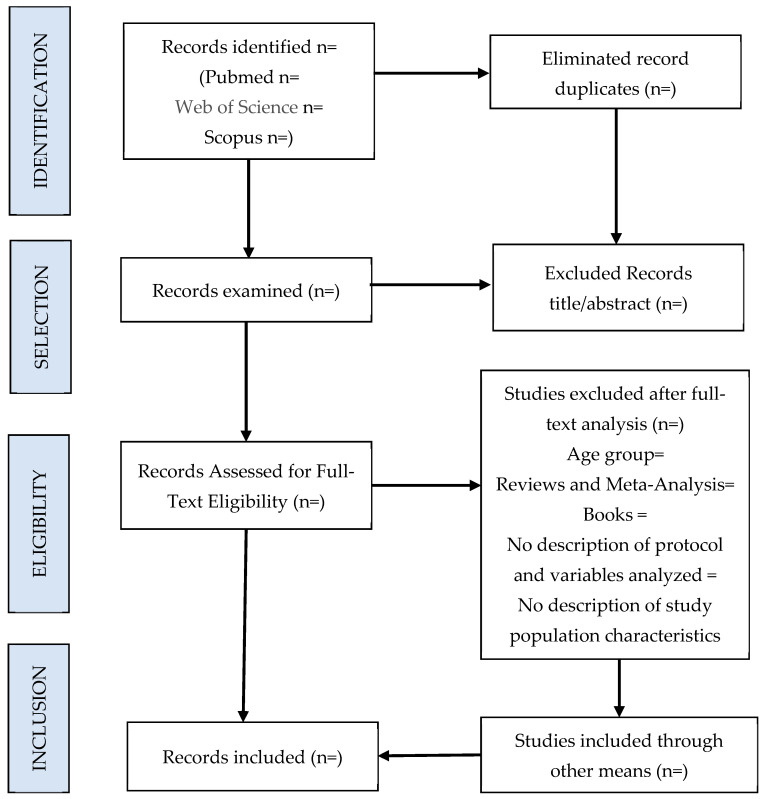
Flowchart of the systematic review search strategy.

Figure 1 shows the planned flowchart of the systematic review proposed in this protocol.

### 2.1. Population

This systematic review will include studies in which the sample is composed of adults aged 18 years or older. “In addition, the study sample of these investigations must suffer or have had an episode of ALBP. Provide a comparison with healthy subjects, provided that the study has a control group. When it comes to cross-sectional or descriptive studies, in some cases a control group approach could be dispensed with. The level of physical activity and participation in training or rehabilitation programs will be monitored. Controlling for both physical activity levels and participation in training and rehabilitation programs will ensure an accurate characterization of the population, considering variables that could affect both pain and HRV, as well as the possibility of analysing if these factors alter the relationship between HRV and ALBP”.

### 2.2. Intervention

Case reports, pilot cohort studies, clinical studies, clinical trials and randomized clinical trials that assess HRV in response to pain will be included (ALBP). The study methodology should include a description of both the protocol and the variables analyzed. The experimental group participants may or may not be undergoing a rehabilitation program and can be active or sedentary. Rehabilitation programs aimed at pain therapies (cryotherapy, physiotherapy, chiropractic, etc.), strength training, postural correction and proprioception will be considered. In cross-sectional studies, a single measure is expected, while longitudinal studies must contain at least one pre-post evaluation, as well as a follow-up if defined in the study methodology.

The variables in each study methodology that are directly related to HRV and ALBP shall be analyzed. In addition, in studies that include rehabilitation programs, it is necessary to examine the characteristics and impact of the interventions in question on the HRV and ALBP variables. In any case, the timing of the HRV measurements will be specified.

### 2.3. Comparator

The inclusion of studies in this review is determined by the methodology and type of population. That is, studies with both cross-sectional and longitudinal designs will be included. In addition, pilot studies will be considered for inclusion. In any case, the study sample must be adults over 18 years of age with ALBP.

When the study methodology does not include a training or rehabilitation program, it is not necessary for the studies to have a control group to be included in the review. This criterion will be followed since, when the studies are descriptive or cross-sectional, it is possible that they only include a single population group. Otherwise, there must be a control group. The control groups should have received lighter and/or more traditional training or therapies than the experimental group or be passive control groups.

This decision is motivated by the need to include and take into account different approaches and perspectives that allow for addressing the relationship between HRV and ALBP in a more comprehensive way. This refers to analyzing different protocols and measurement conditions, comparing between people with different physical activity levels or subjected (or not) to different training or therapies aimed at postural correction, strength training and pain treatment.

### 2.4. Outcomes

Participant Characterization

An analysis of the characteristics of the participants will be presented for each study. First, participant sociodemographic, physical and physiological characteristics must be analyzed in each study. Pain-related outcomes will include the origin of the pain as well as specific characteristics and prognosis.

Regarding the HRV, results will indicate the time and frequency domains assessed (very low frequency “VLF”, low frequency “LF”, high frequency “HF”, total power, Standard deviation of all the normal-to-normal intervals “SDNN”, root mean square successive difference “RMSSD”, percentage (%) of total interval pairs that differ by more than 50 milliseconds “PNN50”, among others). In cases where training or therapy has been conducted, all variables (pain and HRV) shall be controlled before and after the intervention. When the studies include a follow-up period, both the follow-up time and the measurements carried out in this period will be considered. Only one measurement is expected if the study is cross-sectional.

Finally, the results on the relationship between both variables (pain and HRV) will show, on the one hand, the correlation between the two and, on the other, the relevant factors that explain this relationship.

### 2.5. Inclusion and Exclusion Criteria

The study will include adults with acute back pain, preferably with ALBP. The studies can be clinical trials, randomized controlled trials, case reports, pilot cohort studies and clinical studies. Studies that contain some sort of intervention must clearly define the intervention in the methodology section.

Studies that do not clearly and precisely define the characteristics of the study population, the variables analyzed or that do not meet the inclusion criteria described above shall be excluded. People with diseases that could affect HRV will be excluded, such as subjects with cardiovascular diseases or blood pressure alterations. Those who are taking medications that may have a direct effect on blood pressure, heart rate or pain will also be excluded. All studies that are not written in English or Spanish will also be excluded.

### 2.6. Research Question

Is heart rate variability a valid alternative method to measure acute lower back pain?

### 2.7. Literature Search Strategy

The sources used to identify studies for the systematic review will be PubMed, Web of Science (all data bases) and Scopus. The search will be limited to published clinical trials, randomized controlled trials, case reports, pilot cohort studies, clinical studies and reports written in English and Spanish. No time limit is established.

The search will use the Mesh Terms (Sciatica; Autonomic nervous system and Vagus nerve) and non-Mesh Terms (Heart rate variability; Acute low back pain and Acute Low Backaches). All will be combined using the Boolean operators AND and OR as follows: (heart rate variability OR vagus nerve OR autonomic nervous system) AND (acute low back pain OR acute low backaches OR sciatica) (Table 1). Additionally, a snowballing technique will be used (the reference list of an article or the citations of the article can be used to identify additional articles) [28].

### 2.8. Data Extraction and Statistical Analysis Plan

#### 2.8.1. Search and Selection

Firstly, the study search and selection process will be conducted by two reviewers. To this end, the search, inclusion and exclusion criteria described above will be applied, taking article titles and abstracts into account. In cases of disagreement between the reviewers, a third reviewer will examine the studies. A Rayyan directory will be used to save all the selected references.

On this platform, a first reviewer will filter out duplicates. Subsequently, the selection (inclusion and exclusion) of the remaining studies will be carried individually by two independent reviewers (blind on), assigning labels according to the inclusion criteria and the reason of exclusion of each study. Once this second filtering is completed, the status of the review will go to blind off, with the aim of comparing the decisions of the two reviewers. If discrepancies arise, a third reviewer must intervene.

#### 2.8.2. Extraction

Secondly, once the studies have been selected, the following information will be extracted: author names, year of publication, experimental and control group characteristics, pain characteristics, HRV domains, intervention characteristics and relationship between HRV and lower back pain. Cochrane’s Review Manager software (RevMan 5.4) will be used to extract data. Two independent reviewers will extract the data from each study individually. In cases of disagreement or discrepancies between two files, the two reviewers who worked on the file in question must resolve the issue and reach an agreement.

#### 2.8.3. Synthesis and Presentation

The data synthesis strategy will follow the recommendations of Campbell et al. [29].

Studies will be grouped according to the study design and participant characteristics. Study heterogeneity will be controlled by ordering the tables by hypothesized modifiers (study design and sample characteristics). The GRADE (Grading of Recommendations, Assessment, Development and Evaluations) framework will be used to assess certainty. This system allows for establishing a grading of the quality of the evidence (high or low) and the strength of the recommendations (risks/benefits, values and preferences of the participants, consumption of resources or costs) [30]. The data will be presented in tables according to the methodology and final grouping of the results. We will analyze the following moderators: population (healthy participants vs. non-healthy participants); sex (male, female); HRV domains; pain (level, type, location); and study design. A first table will be used to present the data related to the design, participant characteristics, and methodological aspects. A second table will be used to present the studies together with the variables analyzed, including that of HRV. In this sense, a comparative graph will also be presented allowing for the analysis of the percentage of use of HRV domains.

### 2.9. Risk of Bias

Random sequence generation, allocation concealment, blinding of participants and personnel, blinding of outcome assessment, incomplete outcome data, selective reporting and other bias will be assessed. The assessment will be performed at a study level. Cochrane’s Review Manager (RevMan) software will be used for risk of bias analysis.

These seven domains can be grouped into five dimensions [31]. The content and considerations to take into account in each of them are as follows:

Allocation process:

1. Random sequence generation: This domain assesses whether the study employed a randomly generated sequence for assigning participants.
2. Allocation concealment: This domain evaluates the method used to hide the allocation sequence, enabling the determination of whether intervention assignments were planned before or during enrollment.

Blinding process:

3. Blinding of participants and personnel: This domain evaluates the measures, if present, implemented to keep participants and study personnel unaware of the assigned interventions.
4. Blinding of outcome assessment: This dimension assesses if those determining outcome measurements possess knowledge of intervention assignments, which may introduce bias. This section details any measures taken to blind outcome assessors from knowing the participants’ intervention.

Attrition bias:

5. Incomplete outcome data: This domain oversees the availability of comprehensive information on dropouts, exclusions, participant distribution in each intervention group, reasons for dropouts/exclusions and any re-inclusions in the conducted analyses. The absence of outcome data, stemming from attrition or exclusions during the study, increases the risk of biased effect estimates. The term “incomplete outcome data” encompasses both attrition and exclusions, and if an individual participant’s outcome is unavailable, it is labeled as ‘missing’.

Reporting bias:

6. Selective reporting: This domain outlines the likelihood of selectively reporting results and presenting the findings.

Other bias:

7. Other sources of bias: Any significant concerns regarding bias not covered in the other domains should be highlighted here.

Two reviewers will take part in the quality assessment process. In case of disagreement between the two reviewers, a third reviewer will break the tie.

### 2.10. The Status and Timeline of the Study

The search phase is expected to start on the 1 September 2023. The search process is expected to be completed by the 30 November 2023. The document is projected to be completed within a period of approximately 6 months.

## 3. Expected Results

An accurate and early diagnosis of ALBP is especially relevant for determining the most appropriate treatment and improving the quality of life of those who suffer from it as fast as possible. Lower back pain is a complex phenomenon and possibly requires different approaches and diagnostic techniques. Among these methods, HRV could be an interesting technique to consider in this complex process.

This study will search for publications in which HRV is used as a measurement instrument or as a variable to consider in people with ALBP, that is, studies that analyze the relationship between both variables or that use HRV as a method for detecting ALBP. Specifically, it is expected, on the one hand, to determine the efficacy of HRV for detecting ALBP accurately. On the other hand, it is intended to define the most useful HRV domains for the evaluation of ALBP and to establish the steps to follow in the evaluation process taking into account the measurement of HRV.

## 4. Discussion

This systematic review is proposed with the objective of analyzing the relationship between HRV and ALBP, as well as determining the most effective strategy to use HRV as a parameter for evaluating episodes of ALBP. Pain is a sensory, subjective and intrinsic perception of the subject [6]. Musculoskeletal pain is often one of the most disabling types of pain. For instance, lower back pain is the most widespread musculoskeletal problem among populations of all ages. A major consequence of lower back pain is difficulties in performing everyday tasks and work, together with the impact of pain on mental and social health [32,33]. This leads to a disproportionate increase in economic, health and business costs for companies with affected workers [34]. Given that one of the risk factors for chronic lower back pain is repeated ALBP episodes, it is necessary that public health systems prevent and treat this acute pain.

It seems that, in the presence of a musculoskeletal injury, an alteration of the autonomic function occurs due to increased afferent inputs from sensitized nociceptors and other sensory neurons [35]. Given the relationship between pain and the activation of the autonomic nervous system, physiological measurement techniques are currently being used to detect pain [6]. Among these variables, HRV could be an interesting technique to consider in the evaluation and/or identification of ALBP. HVR has been analyzed for both sports performance and health improvement purposes [36,37,38,39]. Regarding the field of health, pain can be considered as a stressful situation for our bodies, and HRV is altered in stressful situations [40,41]. In fact, HRV is related, among other elements, to the functionality of the autonomic nervous system (sympathetic and parasympathetic branches) [1]. This relationship suggests that HRV alterations could be observed in subjects with low pain or subjected to certain movements that increase the joint lumbar spine stress.

To the best of our knowledge, only one systematic review has focused on the relationship between HRV and lower back pain, but it centers on chronic lower back pain [22]. Thus, the present study established the protocol to follow to carry out a systematic review to determine the relationship between HRV and ALBP. The results obtained in this review could provide information on the effectiveness of HRV as an alternative method for measuring ALBP, different methods to detect ALBP using HRV, as well as the possible influence of different physical activity levels and training or rehabilitation programs in the relationship between HRV and ALBP.

### Strengths and Limitations

This review study has certain strengths. Among them, the analysis quality of the studies selected using the Pedro scale and the use of the GRADE system stands out. On the other hand, the novelty of the study is highlighted. The most recent systematic review regarding the relationship between lower back pain and HRV is the one published by Bandeira et al. [22], but it focuses on chronic lower back pain and includes only two studies. To the best of our knowledge, there are no systematic reviews that analyze the relationship between HRV and ALBP. For this reason, this systematic review could provide valuable information for researchers and clinicians on the use of HRV as an additional measurement technique for the diagnosis of ALBP.

This study also has some limitations. First, we must consider the possibility of finding a limited number of studies that evaluate the role of HRV in detecting ALBP and the heterogeneity of the methodologies and samples used in these studies. This could make it difficult to obtain results for comparison. Secondly, the possibility of having few studies with interventions could make it challenging to discern whether the changes in HRV are due to training or improvement in pain. Finally, it is also possible that none of the studies found meet the eligibility criteria, in which case it would be considered an empty review [42]. Therefore, making a first prediction, it is possible that future lines of research should focus on the use of HRV as an instrument for evaluating ALBP. To this end, our review can contribute by helping in the selection of the best HRV domains and measurement protocol.

## 5. Conclusions

In conclusion, this study establishes a systematic review protocol to help identify the relationship between HRV and ALBP. Understanding this relationship could help design early detection or action protocols that alleviate ALBP.

## Figures and Tables

**Table 1:** Search strategy.

Search Strategy	
1	heart rate variability OR vagus nerve OR autonomic nervous system AND
2	acute lower back pain OR acute lower backaches OR sciatica

## Data Availability

Data are contained within the article.
